# Effectiveness of training stop‐smoking advisers to deliver cessation support to the UK national proposed standard versus usual care in Malaysia: a two‐arm cluster‐randomized controlled trial

**DOI:** 10.1111/add.15346

**Published:** 2021-01-20

**Authors:** Lei Hum Wee, Robert West, Guat Hiong Tee, Lena Yeap, Caryn Mei Hsien Chan, Bee Kiau Ho, Komathi Perialathan, Mohamad Haniki Nik Mohamed, Susan Michie, Sarah E. Jackson

**Affiliations:** ^1^ Universiti Kebangsaan Malaysia Kuala Lumpur Malaysia; ^2^ Department of Behavioural Science and Health University College London London UK; ^3^ Ministry of Health Institute for Public Health Malaysia; ^4^ Stats Consulting Sdn. Bhd Malaysia; ^5^ Ministry of Health Bandar Botanic Health Centre Malaysia; ^6^ Ministry of Health Institute of Health Behavioural Research Malaysia; ^7^ International Islamic University Malaysia Malaysia; ^8^ Centre for Behaviour Change University College London London UK

**Keywords:** Effectiveness, Malaysia stop smoking services, randomized controlled trial, smoking cessation, stop smoking services, UK National Centre for Smoking Cessation and Training (UK NCSCT)

## Abstract

**Aims:**

To assess the effectiveness of training stop smoking services providers in Malaysia to deliver support for smoking cessation based on the UK National Centre for Smoking Cessation and Training (NCSCT) standard treatment programme compared with usual care.

**Design:**

Two‐arm cluster‐randomized controlled effectiveness trial across 19 sites with follow‐up at 4‐week, 3‐month, and 6‐month.

**Setting:**

Stop smoking services operating in public hospitals in Malaysia.

**Participants:**

Five hundred and two smokers [mean ± standard deviation (SD), age 45.6 (13.4) years; 97.4% male] attending stop smoking services in hospital settings in Malaysia: 330 in 10 hospitals in the intervention condition and 172 in nine hospitals in the control condition.

**Intervention and comparator:**

The intervention consisted of training stop‐smoking practitioners to deliver support and follow‐up according to the NCSCT Standard Treatment Programme. The comparator was usual care (brief support and follow‐up).

**Measurements:**

The primary outcome was continuous tobacco smoking abstinence up to 6 months in smokers who received smoking cessation treatment, verified by expired‐air carbon monoxide (CO) concentration. Secondary outcomes were continuous CO‐verified tobacco smoking abstinence up to 4 weeks and 3 months.

**Results:**

Follow‐up rates at 4 weeks, 3 months and 6 months were 80.0, 70.6 and 53.3%, respectively, in the intervention group and 48.8, 30.8 and 23.3%, respectively, in the control group. At 6‐month follow‐up, 93 participants in the intervention group and 19 participants in the control group were abstinent from smoking, representing 28.2 versus 11.0% in an intention‐to‐treat (ITT) analysis assuming that participants with missing data had resumed smoking, and 52.8 versus 47.5% in a follow‐up‐only (FUO) analysis. Unadjusted odds ratios (accounting for clustering) were 5.04, (95% confidence interval (CI) = 1.22–20.77, *P* = 0.025) and 1.70, (95% CI = 0.25–11.53, *P* = 0.589) in the ITT and FUO analyses, respectively. Abstinence rates at 4 week and 3 month follow‐ups were significantly higher in the intervention versus control group in the ITT but not the FUO analysis.

**Conclusions:**

On an intention‐to‐treat analysis with missing‐equals‐smoking imputation, training Malaysian stop smoking service providers in the UK National Centre for Smoking Cessation and Training standard treatment programme appeared to increase 6 month continuous abstinence rates in smokers seeking help with stopping compared with usual care. However, the effect may have been due to increasing follow‐up rates.

## Introduction

Stop smoking services that provide a combination of behavioural and pharmacological support have been shown to be effective in increasing success rates in smokers trying to stop smoking [1, 2], but few randomized controlled trials have been undertaken outside high‐income countries. Contextual factors and variation in delivery of stop smoking services may lead to large differences in outcomes of specific treatment services as well as cross‐nationally [2, 3]. To address the variability in stop smoking service delivery within England, the English Department of Health commissioned the National Centre for Smoking Cessation and Training (NCSCT) (www.ncsct.co.uk) to (i) identify the competences required to deliver, manage and commission smoking cessation support; (ii) develop and implement methods of assessment of these competences; and (iii) commission and provide training and continuing support to allow staff to achieve the required level of competence. This led to development of the NCSCT standard treatment programme and evidence‐based training [4] (for details see ‘Intervention’ section). In England this training appears to increased knowledge [5], confidence in skills [6] and success rates of services [7]. This paper reports a trial assessing whether training based on this model, delivered to stop smoking practitioners in Malaysia, a country with a different culture and tobacco control climate, would increase success rates of stop smoking services.

Whether NCSCT training can be applied internationally to boost success rates of stop smoking services in countries other than the United Kingdom has not been tested. As a country with widespread provision of free stop smoking services, Malaysia provided a useful test bed. Different stop smoking services in Malaysia have been shown to have markedly different success rates [8, 9], attributable in some part to differences in the way the treatment is delivered [10]. Providing evidence‐based training to stop smoking, practitioners could help to standardize service delivery and increase practitioners’ knowledge, skills and confidence in supporting smokers to quit [5, 6], thus boosting success rates across the country [7].

In Malaysia, smoking prevalence has been relatively stable at just over 20% for the past three decades, with the most recent national data from 2019 indicating 21.3% of adults were current smokers [11]. There are pronounced gender differences in smoking, with rates approximately 30 times higher among men (43.0%) than women (1.4%) [11], which may reflect differences in the cultural acceptability of smoking for men and women. The most commonly used form of tobacco is manufactured cigarettes (20.1% of the adult population); use of hand‐rolled cigarettes (2.3%) and other smoked tobacco (0.2%) is relatively rare [11]. A high proportion of the Malaysian population face regular exposure to second‐hand smoke in the home (31%), work (27%) and public places (up to 50%) [12]. While steps have been taken in an effort to reduce this, such as the designation of all food and beverage outlets as smoke‐free areas from January 2019 [13], enforcement is lacking, willingness of smokers to comply with tobacco control laws is low (~50%) and the collectivist culture means non‐smokers are typically reluctant to disrupt harmony by exerting their rights to smoke‐free air in public places [14, 15].

The 2015 National Health and Morbidity Survey indicated that, overall, more than half of current smokers (52.3%) in Malaysia made an attempt to quit smoking in the past 12 months, but the rate of quit attempts were higher among younger than older smokers [11]. Stop smoking services in Malaysia are offered within public hospitals and primary care settings free of charge to all smokers who want to quit. Smokers who attend stop smoking services are typically male, middle‐aged, highly educated, highly nicotine‐dependent and motivated to quit for health reasons [16]. Success rates within these services are typically in the region of 30–40% among smokers followed‐up [8, 9, 17, 18], although estimates vary according to study methodology, duration of follow‐up, use of biochemical validation and types of intervention delivered across different settings.

The present study aimed to assess the effectiveness of training stop smoking service providers in Malaysia to deliver a stop smoking service based on an adapted version of the UK NCSCT standard treatment programme compared with usual care to aid 6‐month smoking abstinence. We chose a randomized controlled trial because it was important to be able to attribute any effect to the intervention, and we chose a cluster design because once a stop smoking adviser has been trained in a new method, it cannot be assumed that they will be able to apply usual care to randomly selected clients. Usual care was selected as the comparator in order to evaluate the potential benefit of the enhanced stop smoking service over and above the current offering.

The primary research question (RQ) was:

1. What is the effectiveness of training stop‐smoking practitioners to provide an adapted version of the UK NCSCT standard treatment programme compared with usual care in achieving an increase in the percentage achieving 6‐month continuous tobacco smoking abstinence in smokers who attend stop smoking services in Malaysia?

Secondary RQs were:

1. In the same population, setting and intervention versus comparator for RQ1, what is the effect in achieving an increase in the percentage achieving 4‐week continuous tobacco smoking abstinence?
2. In the same population, setting and intervention versus comparator for RQ1, what is the effect in achieving an increase in the percentage achieving 3‐month continuous tobacco smoking abstinence?

## Method

### Design

This was a two‐arm cluster‐randomized controlled effectiveness trial with random allocation of hospital clinics to 10 hospitals (acting as clusters) in the intervention condition and nine hospitals in the comparator condition. The study protocol and analysis plan were not pre‐registered, but the anonymized data are included in the Supporting information, File S1 and are available for any additional sensitivity analyses other researchers may wish to perform.

### Setting

The study was conducted in public hospital outpatient facilities providing stop smoking services in Malaysia. Recruitment took place between January 2013 and July 2014. In Malaysia, stop smoking services are provided through hospital and primary care settings. At the time the study began, there were 22 major public hospital outpatient facilities that provided stop smoking services.

### Participants

Hospital inclusion criteria were: (i) recorded at least five new smokers each month and (ii) employed at least one dedicated member of staff for managing stop smoking services. Directors from all participating hospitals provided written informed consent.

Stop smoking practitioners inclusion criteria were: (i) health professionals who provided services in stop smoking clinics within eligible hospitals (e.g. medical officers, pharmacists, health education officers, nurses and medical assistants). Practitioners provided verbal consent face‐to‐face when they attended the briefing and training.

Potential participants were identified by stop smoking, practitioners in participating hospitals, who were asked to recruit all eligible smokers seen in routine stop smoking services. Smokers could access the stop smoking services by physician‐ or self‐referral.

Participants inclusion criteria were: (i) adult (≥ 18 years), (ii) current smokers who were (iii) seeking assistance from stop smoking services for the first time or after more than 6‐month since a past attempt to quit with stop smoking service support, (iv) willing to participate in the study and (v) able to provide consent. There were no additional exclusion criteria.

Stop smoking service staff screened service users for eligibility. Eligible smokers were provided with information about the study and those who agreed to participate provided written informed consent. Participants were not provided with any financial compensation for taking part in the study.

### Sample size determination

The intended sample size was decided using a priori power calculation and Epi Info version 6 software. The odds ratio (OR) effect size parameter was used and the effect size tested for was OR = 2 or greater with a two‐tailed alpha of 0.05 at 80% and a projected success rate of 20% in the intervention condition and 10% in the control condition. This led to a target sample size of 240 in the Intervention group and 240 in the comparator group, assuming loss to follow‐up of 20%. The power calculation did not involve correction for clustering assuming a negligible design effect.

### Procedures

#### Identification and recruitment of study sites

All public hospitals in Malaysia that provided stop smoking services were contacted about the study and evaluated for inclusion. The director of each hospital was contacted by members of the research team from the Health Behavioural Research Institute, National Institute of Health Malaysia. Those who expressed interest and met the inclusion criteria were sent a formal letter signed by the Director of the Health Behavioural Research Institute providing further information about the study. Interested hospitals were requested to provide written feedback on (i) the number of smokers treated in the past 5 years and (ii) the use of carbon monoxide (CO) analysers at each patient visit. All communication was in the national language of Malaysia (Bahasa Malaysia).

Twenty out of 22 public hospitals met the hospital inclusion criteria and were recruited and randomized. The two hospitals that did not meet the inclusion criteria reported too few clinic attendees. Thus, this study had representation from most hospitals offering quit smoking services in Malaysia. One hospital dropped out after randomization but before participant enrolment because trained staff left the stop smoking service. Therefore, a total of 19 hospitals remained in the trial.

#### Randomization

To allocate 10 hospitals to the intervention condition and 10 to the control condition, a set of 10 1 s and 10 2 s was created on pieces of paper and jumbled up so the numbers could not be seen. One co‐investigator, observed by other members of the research team, then drew pieces of paper one at a time without replacement, going down the list of hospitals allocating the 1 s to intervention and 0 s to control. One hospital in the control condition dropped out (see above), leaving 10 hospitals in the intervention condition and nine in the control condition. The hospitals in each condition did not differ significantly in the total number of smokers attending their stop smoking services in the 6 months prior to the start of the trial [mean ± standard deviation (SD) = 44.6 (30.37) in intervention hospitals versus 42.6 (14.99) in control hospitals, *P* = 0.661]. Neither the researchers nor hospitals were blinded to group allocation.

#### Data collection and measures

In both the intervention and comparator groups, stop smoking practitioners were briefed on the research procedures in the same time‐frame. Three field visits were undertaken by the research team to promote fidelity of intervention delivery and data collection procedures, but no assessment of fidelity of intervention delivery was undertaken.

Data were collected via self‐report questionnaires at participants’ first visit to the stop smoking services (pre‐quit) and at 4 weeks and 3 and 6 months post‐quit date. The questionnaires were adapted and modified from a previous study based in a similar setting [8, 17], and piloted with 30 patients at the Tanglin Health Centre stop smoking service, Kuala Lumpur. Questionnaires were available in Bahasa Malaysia and English. Stop smoking service staff provided assistance for those who needed help answering the questionnaires. Where possible, telephone interviews were carried out for smokers who did not attend follow‐up appointments, but attendance for assessment of CO was required (see below) if participants were to be recorded as abstinent.

The pre‐quit questionnaire included assessment of socio‐demographic characteristics, smoking characteristics and history, health status and motives for quitting (see Tables 1 and 2). The 4‐week, 3‐month and 6‐month post quit‐date follow‐ups assessed smoking status and a range of variables designed to help identify reasons for relapse (not reported here).

**Table 1:** Baseline socio‐demographic characteristics

Characteristics	Control (*n* = 172)	Intervention (*n* = 330)	Overall (*n* = 502)	*P* ^*^
Age (years), mean ± SD	45.6 ± 14.1	45.7 ± 13.1	45.6 ± 13.4	0.981
Age (years), % (*n*)				
< 25	7.0 (12)	5.8 (19)	6.2 (31)	0.494
25–34	19.8 (34)	16.1 (53)	17.3 (87)	
35–44	16.9 (29)	22.7 (75)	20.7 (104)	
45–54	28.5 (49)	30.0 (99)	29.5 (148)	
> 54	27.9 (48)	25.5 (84)	26.3 (132)	
Gender, % (n)				
Male	99.4 (171)	96.4 (318)	97.4 (489)	0.041
Female	0.6 (1)	3.6 (12)	2.6 (13)	
Ethnicity, % (*n*)				
Malay	79.1 (136)	56.1 (185)	63.9 (321)	<0.001
Chinese	12.2 (21)	20.9 (69)	17.9 (90)	
Indian	8.7 (15)	12.7 (42)	11.4 (57)	
Other	0.0 (0)	10.3 (34)	6.8 (34)	
Highest level of education, % (*n*)				
None/primary school	18.6 (32)	17.3 (57)	17.7 (89)	0.739
Lower/higher secondary school	57.0 (98)	55.2 (182)	55.8 (280)	
Pre‐university/matriculation/A‐level/cert/diploma/degree	24.4 (42)	27.6 (91)	26.5 (133)	
Marital status, % (*n*)				
Married	80.8 (139)	81.2 (268)	81.1 (407)	0.758
Unmarried (single/divorced)	19.2 (33)	18.5 (61)	18.7 (94)	
Missing	0 (0)	0.3 (1)	0.2 (1)	
Occupation, % (*n*)				
Government	22.1 (38)	16.7 (55)	18.5 (93)	0.001
Private	22.1 (38)	39.4 (130)	33.5 (168)	
Self‐employed	30.8 (53)	27.0 (89)	28.3 (142)	
Other (pensioner/student/housewife/not working)	25.0 (43)	17.0 (56)	19.7 (99)	
Shift work, % (*n*)				
Yes	11.6 (20)	23.0 (76)	19.1 (96)	0.012
No	88.4 (152)	77.0 (254)	80.9 (406)	
Work stress level, % (*n*)				
Very stressed	1.7 (3)	4.5 (15)	3.6 (18)	0.191
Stressed	23.3 (40)	27.9 (92)	26.3 (132)	
Less stress	28.5 (49)	34.8 (115)	32.7 (164)	
No stress	37.2 (64)	25.8 (85)	29.7 (149)	
Not sure	9.3 (16)	7.0 (23)	7.8 (39)	
Perceived health problems, % (*n*)				
Yes	77.3 (133)	81.5 (269)	80.1 (402)	0.606
No	22.7 (39)	18.5 (61)	19.9 (100)	

SD = standard deviation.

^*^ Accounts for clustering.

**Table 2:** Baseline smoking characteristics

Characteristics	Control (*n* = 172)	Intervention (*n* = 330)	Overall (*n* = 502)	*P* ^*^
Age of smoking initiation (years), mean ± SD	17.1 ± 4.0	17.5 ± 4.6	17.4 ± 4.4	0.382
Time to first cigarette after waking (minutes), % (*n*)				
≤ 5	21.5 (37)	22.1 (73)	21.9 (110)	0.072
6–30	40.1 (69)	32.4 (107)	35.1 (176)	
31–60	15.1 (26)	28.5 (94)	23.9 (120)	
> 60	23.3 (40)	17.0 (56)	19.1 (96)	
No. of cigarettes smoked per day				
Number	172	329	501	
Mean ± SD	16.2 ± 10.3	16.5 ± 10.6	16.4 ± 10.5	0.814
Median (IQR)	15 (10, 20)	15 (10, 20)	15 (10, 20)	
(min, max)	(2, 60)	(1, 100)	(1, 100)	
Urge to smoke in the past 7 days, % (*n*)				
Not at all	10.5 (18)	7.3 (24)	8.4 (42)	0.021
A little of the time	9.3 (16)	20.3 (67)	16.5 (83)	
Some of the time	41.9 (72)	35.8 (118)	37.8 (190)	
Almost all the time	23.3 (40)	24.5 (81)	24.1 (121)	
All the time	15.1 (26)	12.1 (40)	13.1 (66)	
Smoked at home, % (*n*)				
Yes	67.4 (116)	66.1 (218)	66.5 (334)	0.766
No	32.6 (56)	33.9 (112)	33.5 (168)	
Exposed to second‐hand smoke at home, % (*n*)				
Yes	21.5 (37)	25.2 (83)	23.9 (120)	0.472
No	78.5 (135)	74.8 (247)	76.1 (382)	
Method of quitting, % (*n*)				
Abrupt cessation	46.5 (80)	57.6 (190)	53.8 (270)	0.300
Gradual cessation^a^	53.5 (92)	42.4 (140)	46.2 (232)	
Carbon Monoxide (CO) level				
Number	148	313	461	
Mean ± SD	8.0 ± 5.4	11.1 ± 7.3	10.1 ± 6.9	0.094
Median (IQR)	7 (4, 11)	11 (5, 15)	9 (5, 14)	
Range (min, max)	[1, 25]	(1, 46)	(1, 46)	
Motives for smoking, % (*n*)				
Staying calm in stressful situations	59.9 (103)	49.4 (162)	53.0 (265)	0.370
Keeping you from getting too fat	39.0 (67)	28.7 (94)	32.2 (161)	0.180
Helping you to concentrate and stay alert	11.0 (19)	4.9 (16)	7.0 (35)	0.059
Stopping you from being bored	41.3 (71)	24.4 (80)	30.2 (151)	0.005
Enjoying being with friends	53.5 (92)	66.8 (219)	62.2 (311)	0.138
Feeling better when bad things happen	33.1 (57)	34.5 (113)	34.0 (170)	0.852
Feeling uncomfortable if not smoking	44.8 (77)	36.3 (119)	39.2 (196)	0.172
Smoking is an enjoyment	23.8 (41)	19.8 (65)	21.2 (106)	0.362
Others	1.7 (3)	1.8 (6)	1.8 (9)	0.938
Reasons to quit smoking, % (*n*)				
Pressure from friends and family	13.4 (23)	14.6 (48)	14.2 (71)	0.865
Concern about personal health/illness	83.1 (143)	77.5 (255)	79.4 (398)	0.487
Concern about health of other family members	52.9 (91)	33.1 (109)	39.9 (200)	0.114
Doctor's/health professional advice and orders	61.0 (105)	72.9 (240)	68.9 (345)	0.259
Cost of cigarettes increasing (financial)	26.7 (46)	16.7 (55)	20.2 (101)	0.138
Restriction on smoking in public places	15.1 (26)	5.5 (18)	8.8 (44)	0.022
Social stigma (seen in negative light)	14.5 (25)	5.8 (19)	8.8 (44)	0.093
Religion/beliefs	20.9 (36)	9.1 (30)	13.2 (66)	0.040
Others	2.3 (4)	1.5 (5)	1.8 (9)	0.460
Confidence to stop smoking completely, % (*n*)				
Very confident	41.9 (72)	37.6 (124)	39.0 (196)	< 0.001
Quite confident	46.5 (80)	51.5 (170)	49.8 (250)	
Not very confident	8.1 (14)	8.5 (28)	8.4 (42)	
Not at all confident	0.0 (0)	0.3 (1)	0.2 (1)	
Not sure	3.5 (6)	2.1 (7)	2.6 (13)	
Motivation to stop smoking completely, % (*n*)				
Very strong	38.4 (66)	36.7 (121)	37.3 (187)	0.960
Quite strong	48.8 (84)	50.9 (168)	50.2 (252)	
Not strong	9.9 (17)	10.0 (33)	10.0 (50)	
Not sure	2.3 (4)	1.8 (6)	2.0 (10)	
Missing	0.6 (1)	0.6 (2)	0.6 (3)	

SD = standard deviation; IQR = interquartile range.

^*^ Accounts for clustering.

^a^ Aiming to reduce the number of cigarettes smoked per day over a period of time to 0.

The primary outcome was continuous tobacco smoking abstinence at 6 months, assessed by self‐report at the 6‐month follow‐up verified at the 6‐month by an expired‐air CO concentration of < 10 parts per million (p.p.m.). Secondary outcomes were continuous tobacco smoking abstinence at 4 weeks and 3 months, assessed by self‐report verified by an expired‐air CO concentration of < 10 p.p.m.

### Intervention and comparator conditions

Participating stop smoking practitioners in each group were advised to conduct at least six sessions plus an additional two follow‐ups, as follows:

Session 1: pre‐quit assessment (1 or 2 weeks prior to quit date)
Session 2: quit date
Session 3: 1 week post‐quit date
Session 4: 2 weeks post‐quit date
Session 5: 3 weeks post‐quit date
Session 6: 4 weeks post‐quit date (4‐week follow‐up)
3‐month follow‐up
6‐month follow‐up

For the purpose of this study, stop smoking practitioners in both groups were asked to set a target quit date for each participating smoker. Data on the number of sessions participants attended and use of medication were not recorded.

Stop smoking practitioners were advised to make up to three telephone calls to participants who did not attend the follow‐up appointments and complete the relevant assessment measures via telephone. Participants who did not provide a response after three attempted phone calls were considered to be lost to follow‐up.

There was no assessment of hospital‐ or practitioner‐level differences at baseline; for example, the level of ‘usual’ training provided to stop smoking practitioners, or existing competences and skills.

#### Intervention

For the purpose of this study, practitioners allocated to the intervention condition were asked to stop their usual practice (see ‘Comparator’ section below for details) and follow the NCSCT model.

NCSCT training focused on knowledge‐ and skills‐based competences required to deliver behavioural and pharmacological support for smoking cessation based on guidance documents and randomized controlled trials published in Cochrane Reviews [19]. Stop smoking services manuals were coded for these competences and a subset of 16 competences were found to be associated with short‐term success rates of stop smoking services [20, 21].

Informed by this work on competences for the delivery of stop smoking support, a training programme was developed for this study. Knowledge‐based competences were trained through an on‐line training and assessment programme (http://www.ncsct.co.uk/pub_training.php). Using text, videos and assessment questions, it addressed smoking in the population, smoking and health, why people smoke and find it hard to stop, the process of how smokers manage to stop, effective ways to help people stop smoking, medication use and how to plan and deliver a programme of support. Trainees determined how much time they spent on the training, however the average time for the training in the UK was 2.5 hours [5]. Complementing this, training in behavioural support (skills‐based competences) occurred in 2‐day, face‐to‐face courses in groups of 20–30 practitioners. Each course followed a detailed manual and was led by two trainers, who were experienced practitioners. The behaviour change techniques delivered to smokers in the standard treatment plan have been described in detail elsewhere [7]. All participating practitioners completed all the training modules.

Several amendments were made to the NCSCT training by a local panel of smoking cessation experts to adapt it to the Malaysian context. First, the evidence base presented to trainees was based on Malaysian smoking data. Secondly, scenarios and case studies were based on the local setting, such as using examples of second‐hand smoke exposure in eateries or at home, which is common in Malaysia. Thirdly, bupropion was omitted because it was not available in Malaysian stop smoking services at the time of the study. Finally, the training was translated and validated to Bahasa Malaysia. The translated module was sent for independent expert review. Two UK‐based members of the research team (R.W. and S.M.) with extensive experience in evidence‐based behaviour change and smoking cessation interventions and knowledge of the NCSCT standard treatment programme conducted a workshop to train a team of local smoking cessation experts in delivering the NCSCT training to stop smoking practitioners.

#### Comparator

In Malaysia, stop smoking services practitioners are traditionally trained to follow the stages of change model [22] and 5As and 5Rs brief tobacco intervention [23]. Thus, usual care involved assessing smokers in relation to their stage of change and offering advice and support tailored to the stage of change the smoker was in [22]. Stop smoking practitioners were briefed about the research procedures in a group, provided with a brief update on the current standard of stop smoking services in Malaysia [8], and reminded of the importance of encouraging smokers to adhere to prescribed medications. The duration of time trainees spent on the training was not recorded.

### Statistical analysis

The data were analysed using Stata version 15. We used cluster‐adjusted independent *t*‐tests (using the clttest command) for continuous variables and χ^2^ tests (using the clchi2 command) for categorical variables to assess the difference between the intervention and comparator groups on baseline measures. Outcomes were compared using multi‐level logistic regression analyses (using the melogit command), taking account of cluster. We originally constructed two models for each outcome, one unadjusted and one adjusted for all baseline variables. The adjusted models produced results with wide confidence intervals, probably attributable to the large number of covariates combined with a low outcome rate. Thus, following peer review, we added an additional model which controlled for a more streamlined selection of covariates that might plausibly influence outcomes [age, sex, education, perceived health problems and quit method (gradual versus abrupt)].

Sensitivity of the results was checked using generalized estimating equations (GEEs, using the xtgee command) with site as the random clustering parameter. Two approaches to address missing values were used: (1) intention‐to‐treat (ITT) with smokers who were lost to follow‐up retained in the analyses and classified as continuing smokers (missing‐equals‐smoking imputation), as recommended in the Russell Standard [24] based on the assumption that missing outcomes are not missing at random and (2) follow‐up only (FUO) in which only smokers who responded to the 6‐month follow‐up were included in the analysis, based on the assumption that missing outcomes are missing completely at random within each condition. We opted for a FUO analysis rather than multiple imputation, because follow‐up rates were low, baseline characteristics would not accurately model success rates and analysing complete cases is the only method that fully takes account of the effect of the intervention on follow‐up rates. ORs and 95% confidence intervals (CIs) were computed, together with *P*‐values. We also assessed whether there was a bias resulting from different numbers of smokers being recruited into intervention versus control conditions. To do this we used a Mann–Whitney test to compare the ratio of the number of recruited participants to the total number of smokers attending stop smoking services in the 6‐month prior to the start of the trial in the intervention versus control hospitals.

#### Ethics approval and consent to participate

This study was approved by the Medical Research Ethics Committee, the Ministry of Health, Malaysia (NMRR 11–11–906‐10 630) and selected hospital directors before the study. All participants provided full informed consent.

## Results

A total of 502 participants were recruited and completed the baseline assessment. In the 6 months prior to the study, the mean throughput of smokers through services was 44.6 (range = 20–120) smokers per hospital in the intervention group and 42.6 (range = 20–68) smokers per hospital in the control group. On average, the rate of recruitment during the study relative to past 6‐month throughput did not differ significantly between intervention and control stop smoking services (*P* = 0.243). However, there was substantial variation between sites, with recruitment as low as 8% of past‐6‐month throughput in one intervention and one control hospital and as high as 250% in two intervention hospitals (Supporting information, File S2, Table S1).

**Figure 1:**
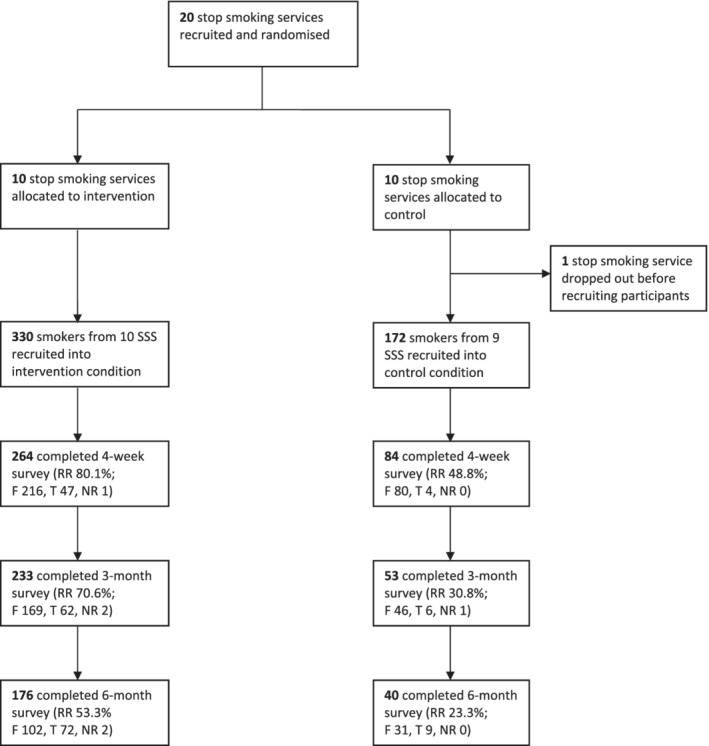
Consolidated Standards of Reporting Trials (CONSORT) flow‐chart. SSS = stop smoking services; RR = response rate; F = number of participants who provided information face‐to‐face; T = number of participants who provided information via telephone; NR = number of participants for whom the mode of information provision was not recorded

Figure 1 shows the numbers allocated to each group and followed‐up, Tables 1 and 2 summarize baseline socio‐demographic and smoking characteristics, respectively. Compared with the control group, a higher proportion of participants in the intervention group were females and non‐Malays, and more reported shift work and work‐related stress. Participants in the intervention group were more likely to smoke within 30 minutes of waking, reported less frequent urges to smoke and had a higher mean CO level.

Response rates to the 4‐week, 3‐month and 6‐month follow‐ups were 80.0, 70.6 and 53.3%, respectively, in the intervention group and 48.8, 30.8 and 23.3%, respectively, in the control group (Fig. 1). The overall follow‐up rate (i.e. the proportion of participants who provided data at all three follow‐ups) was higher in the intervention group than the control group (52.4 versus 23.3%; OR = 7.29, 95% CI = 1.65–32.10, *P* = 0.009). In addition, at each time‐point, a higher proportion of follow‐up assessments were completed via telephone in the intervention group (17.8, 26.6 and 40.9%, respectively) than in the control group (4.8, 11.3 and 22.5%, respectively; Fig. 1).

Table 3 summarizes results relating to the primary and secondary outcomes. At 6‐month follow‐up, 93 participants in the intervention group and 19 participants in the control group reported 6‐month (CO‐verified) continuous abstinence from smoking. In the ITT analysis, this equated to an abstinence rate of 28.2% in the intervention group and 11.0% in the control group; a significant difference (unadjusted OR = 5.04, 95% CI = 1.22–20.77, *P* = 0.025). However, when we took account of differential follow‐up rates in the FUO analysis, the difference between the groups was smaller and not statistically significant (52.8 versus 47.5% respectively; unadjusted OR = 1.70, 95% CI = 0.25–11.53, *P* = 0.589).

**Table 3:** Unadjusted and adjusted logistic regression analyses of treatment effect on CO‐verified continuous abstinence at 4 weeks, 3 months and 6 months

	CO verified continuous abstinence, % (*n*/*N*)		Unadjusted model				Minimally adjusted model^a^				Fully adjusted model^b^			
Analysis	Control	Intervention	OR	95% CI	*P*	ICC	OR	95% CI	*P*	ICC	OR	95% CI	*P*	ICC
Intention‐to‐treat														
4 weeks	35.5 (61/172)	47.0 (155/330)	2.33	1.11–4.86	0.025	0.099	2.52	1.04–6.11	0.042	0.155	4.94	1.48–16.50	0.009	0.207
3 months	19.8 (34/172)	43.0 (142/330)	7.63	2.04–28.57	0.003	0.282	9.89	1.97–49.58	0.005	0.635	42.72	2.57–710.52	0.009	0.641
6 months	11.0 (19/172)	28.2 (93/330)	5.04	1.22–20.77	0.025	0.312	5.44	1.14–25.88	0.033	0.363	17.19	1.15–257.67	0.040	0.629
Follow‐up only														
4 weeks	72.6 (61/84)	58.7 (155/264)	0.69	0.25–1.89	0.471	0.152	0.56	0.18–1.75	0.320	0.194	0.53	0.12–2.45	0.420	0.227
3 months	64.2 (34/53)	60.9 (142/233)	1.80	0.37–8.70	0.467	0.294	1.68	0.25–11.38	0.595	0.400	3.75	0.14–97.59	0.427	0.633
6 months	47.5 (19/40)	52.8 (93/176)	1.70	0.25–11.53	0.589	0.393	1.62	0.23–11.62	0.628	0.396	3.21	0.01–1820.05	0.719	0.868

CO = carbon monoxide; OR = odds ratio; CI = confidence interval; ICC = intraclass correlation.

^a^ Adjusted for age, sex, education, perceived health problems, and quit method.

^b^ Adjusted for age, sex, ethnicity, education, marital status, occupation, shift work, work stress, perceived health problems, age of smoking initiation, time to first cigarette after waking, cigarettes smoked per day, urges to smoke, smoking at home, exposure to second‐hand smoking at home, quit method, baseline CO, motives for smoking, reasons for quitting, confidence to stop smoking completely and motivation to stop smoking completely.

All models (unadjusted and adjusted) account for clustering.

Consistent with the 6‐month abstinence results, the ITT analysis indicated that abstinence rates at 4‐week and 3‐month follow‐ups were significantly higher in the intervention group compared with the control group, but the FUO analysis showed no significant difference in abstinence rates at either time‐point (Table 3, unadjusted models).

After adjustment for socio‐demographic and smoking characteristics, abstinence rates remained significantly higher at each follow‐up point in the intervention group compared with the control group in the ITT analysis, but did not differ significantly at any follow‐up point in the FUO analysis (Table 3, adjusted models).

GEEs produced a very similar pattern of results, although effect sizes were smaller and their 95% CIs were narrower (Supporting information, File S2, Table S2). For the primary outcome of 6‐month continuous abstinence, the unadjusted OR was 3.84 (95% CI = 1.29–11.44, *P* = 0.016) in the ITT analysis and 1.42 (95% CI = 0.38–5.27, *P* = 0.601) in the FUO analysis.

## Discussion

The results provided qualified support for the hypothesis that the application of an adapted version of UK NCSCT standard treatment programme would boost success rates of stop smoking services in Malaysia. In the ITT analysis, which presumed quitters lost to follow‐up had resumed smoking, the UK NCSCT standard treatment programme produced higher biochemically verified abstinence rates than usual care 6 months after the target quit date. However, in the FUO analysis, which excluded all participants who were lost to follow‐up, the difference between the conditions was not significant. Similar results were observed at 4‐week and 3‐month follow‐ups and after adjustment for socio‐demographic and smoking characteristics measured at baseline.

The fact that an intervention effect was not clearly established in the FUO analyses may, in part, result from reduced statistical power arising from a combination of a higher than expected design effect and reduced numbers of analysed participants. However, it also allows for the possibility that bias may have been introduced from differential loss to follow‐up between the groups. While it is common practice to use an ITT approach in smoking cessation trials [24], this can bias effect sizes upwards if the intervention condition leads to higher follow‐up rates than the control condition. In the present study, follow‐up in the control condition was approximately half that in the intervention condition. Relevant to this is the finding that follow‐up rates by telephone were higher in hospitals in the intervention condition than the comparator condition. Given that attendance in person was required to confirm abstinence, all participants who were followed‐up by telephone were classified as continuing smokers, so the higher telephone follow‐up rate in the intervention condition may have bias‐observed abstinence rates downwards relative to the comparator condition.

Whether the higher telephone follow‐up rate was due to greater effort on behalf of the service providers in the intervention versus the control condition or participants in the intervention condition being more receptive to follow‐up attempts made via telephone is unclear. Experience with clinical trials has shown that smokers who relapse are less likely to respond to follow‐up [24], but we cannot rule out that the ITT results were due to the lower follow‐up rate in the control condition, rather than the lower abstinence rate. It may be expected that the true percentage point difference and ORs lie somewhere between the estimates provided by these two methods [25].

The fact that similar results were observed after adjustment for baseline variables that are predictive of successful cessation adds confidence that the results were not due to smokers who found it easier to stop being more likely to be followed‐up in the intervention condition.

Strengths of this study include the nation‐wide coverage of stop smoking services in public hospitals throughout Malaysia and biochemical verification of abstinence from smoking via CO testing. There were also several limitations. First, the low follow‐up rate and different follow‐up rates in the intervention and control conditions are an important limitation. The high intraclass correlation coefficients indicated that a large proportion of the variance in abstinence was accounted for by the clustering of participants within hospitals. This led to much wider CIs around the effect size than was expected. Thus, while the FUO analysis OR of 1.7 for the primary outcome was close to what would have been expected from prior studies, it fell a long way short of statistical significance. Secondly, there was no baseline assessment of usual treatment within hospitals or practitioner skills and competences. Substantial variability in success rates among different stop smoking services in Malaysia has previously been documented [8, 9]. As a result, we cannot be sure that stop smoking practitioners randomized to the two conditions were equivalent. Thirdly, process data were not collected, limiting interpretation of results. Fidelity of intervention delivery was not assessed, so we were not able to determine the extent to which the practitioners in the intervention condition were successful in delivering stop smoking support according to the NCSCT standard treatment programme, or the extent to which those in the intervention and control conditions actually conducted all the prescribed sessions and follow‐up assessments. Differences in fidelity across sites could have influenced both rates of quit success and follow‐up rates and contributed to the large clustering effects within hospitals. Additionally, no information was recorded on the specific ways in which the NCSCT training changed practitioner practice, for example, in the methods used, medications offered or phrasing of advice. Thus, it is not clear how the intervention increased rates of cessation (e.g. was it through increased use of pharmacological support or differences in the skill with which treatment was delivered?). Further research involving recorded treatment sessions and/or qualitative interviews with practitioners could provide useful insights.

## Conclusions

On an intention‐to‐treat analysis (counting participants lost to follow‐up as having relapsed), training stop smoking practitioners to deliver the UK NCSCT standard treatment programme appeared to be more effective than usual care in helping smokers attending hospital‐based stop smoking treatment programmes in Malaysia to achieve smoking cessation. However, the effect may have been due to differences in follow‐up rates.

## Clinical trial registration

None.

## Declaration of interests

R.W. has undertaken research and consultancy for and received travel funds and hospitality from manufacturers of smoking cessation medications (Pfizer, GlaxoSmithKline and Johnson and Johnson). All authors declare no financial links with tobacco companies or e‐cigarette manufacturers or their representatives.

## Funding Information

This study was funded by the Institute for Health Behavioural Research, National Institutes of Health, Ministry of Health Malaysia (NMRR 11–11–906‐10630). Cancer Research UK supported and supplied metadata and S.E.J.’s salary (C1417/A22962). The funders had no final role in the study design; in the collection, analysis and interpretation of data; in the writing of the report; or in the decision to submit the paper for publication.

## Supporting information

**Data S1** Supporting Information.

**Table S1** Rate of recruitment relative to reported throughput of smokers in the 6 months prior to the study
**Table S2** Unadjusted and adjusted generalised estimating equations of treatment effect on CO verified continuous abstinence at 4 weeks, 3 months and 6 months
**Table S3** Predictors of response to 6‐month follow‐up: multivariable logistic regression.

## References

[1] Cahill K., Stevens S., Perera R., Lancaster T. Pharmacological interventions for smoking cessation: an overview and network meta‐analysis. Cochrane Database Syst Rev 2013; 5: CD009329. 10.1002/14651858.CD009329.pub2 PMC8406789 23728690

[2] West R., May S., West M., Croghan E., McEwen A. Performance of English stop smoking services in first 10 years: analysis of service monitoring data. BMJ 2013; 347: f4921. 23963106 10.1136/bmj.f4921

[3] Brose L. S., West R., McDermott M. S., Fidler J. A., Croghan E., McEwen A. What makes for an effective stop‐smoking service? Thorax 2011; 66: 924–926. 21709164 10.1136/thoraxjnl-2011-200251

[4] National Centre for Smoking Cessation and Training . Standard Treatment Programme [internet]. [cited 2019 May 21]. Available at: https://www.ncsct.co.uk/shopdisp_a‐standard‐treatment‐programme‐for‐smoking‐cessation.php (accessed 21 May 2019).

[5] Brose L. S., West R., Michie S., Kenyon J. A. M., McEwen A. Effectiveness of an online knowledge training and assessment program for stop smoking practitioners. Nicotine Tob Res 2012; 14: 794–800. 22249686 10.1093/ntr/ntr286

[6] Brose L. S., West R., Michie S., McEwen A. Evaluation of face‐to‐face courses in behavioural support for stop smoking practitioners. J Smok Cessat 2012; 7: 25–30.

[7] Brose L. S., West R., Michie S., McEwen A. Changes in success rates of smoking cessation treatment associated with take up of a national evidence‐based training programme. Prev Med 2014; 69: 1–4. 25152508 10.1016/j.ypmed.2014.08.021

[8] Wee L. H., Shahab L., Bulgiba A., West R. Stop smoking clinics in Malaysia: characteristics of attendees and predictors of success. Addict Behav 2011; 36: 400–403. 21195553 10.1016/j.addbeh.2010.11.011

[9] Fai S. C., Yen G. K., Malik N. Quit rates at 6 months in a pharmacist‐led smoking cessation service in Malaysia. Can Pharm J 2016; 149: 303–312. 10.1177/1715163516662894 PMC5032936 27708676

[10] Ezat W. P. S., Selahuddeen A. A., Aljunid S. M., Zarihah Z. Patterns and predictors of smoking cessation among smokers attending smoking cessation clinics in peninsular Malaysia. J Commun Health 2008; 14: 17–23.

[11] Institute for Public Health . National Health and Morbidity Survey 2015. Report on smoking status among Malaysian adults. Kuala Lumpur, Malaysia: Ministry of Health Malaysia; 2015.

[12] Institute for Public Health . National Health and Morbidity Survey 2019. Non‐communicable diseases, healthcare demand, and health literacy: Key Findings [internet]. Kuala Lumpur, Malaysia: Ministry of Health Malaysia; 2019. Available at: http://iptk.moh.gov.my/images/technical_report/2020/4_Infographic_Booklet_NHMS_2019_‐_English.pdf (accessed September 2020).

[13] Attorney General's Chambers . Control of Tobacco Product (Amendment) Regulations 2018. reg. 2, P.U. (A) 329, Federal Government Gazette. Putrajaya. Malaysia: Attorney General's Chambers; 2018, pp. 4–5. Available at: https://www.tobaccocontrollaws.org/files/live/Malaysia/Malaysia%20‐%20TC%20Amdt.%20Regs%202018.pdf

[14] Yong H., Foong K., Borland R., Omar M., Hamann S., Sirirassamee B., *et al*. Support for and reported compliance among smokers with smoke‐free policies in air‐conditioned hospitality venues in Malaysia and Thailand: findings from the international tobacco control Southeast Asia survey. Asia Pac J Public Health 2010; 22: 98–109. 20032039 10.1177/1010539509351303 PMC4411242

[15] Li X., Gao J., Zhang Z., Wei M., Zheng P., Nehl E. J., *et al*. Lessons from an evaluation of a provincial‐level smoking control policy in Shanghai, China. PLOS ONE [internet] 2013; 8 [cited 2020 Sep 29]. Available at: https://www.ncbi.nlm.nih.gov/pmc/articles/PMC3769237/ (accessed 29 September 2020). 10.1371/journal.pone.0074306 PMC3769237 24058544

[16] Wee L., West R., Mariapun J., Chan C., Bulgiba A., Peramaiah D., *et al*. Should the threshold for expired‐air carbon monoxide concentration as a means of verifying self‐reported smoking abstinence be reduced in clinical treatment programmes? Evidence from a Malaysian smokers' clinic. Addict Behav 2015; 47: 74–79. 25889913 10.1016/j.addbeh.2015.03.021

[17] Wee L. H., West R., Bulgiba A., Shahab L. Predictors of 3‐month abstinence in smokers attending stop‐smoking clinics in Malaysia. Nicotine Tob Res 2011; 13: 151–156. 21186253 10.1093/ntr/ntq221

[18] Lim K. H., Heng P. P., Nik Mohamed M. H., Teh C. H., Mohd Yusoff M. F., Ling J. M. Y., *et al*. Prevalence and factors associated with attempts to quit and smoking cessation in Malaysia. Asia Pac J Public Health 2019; 31: 22S–31S. 31802718 10.1177/1010539519874944

[19] Michie S., Churchill S., West R. Identifying evidence‐based competences required to deliver behavioural support for smoking cessation. Ann Behav Med 2011; 41: 59–70. 20936389 10.1007/s12160-010-9235-z

[20] West R., Walia A., Hyder N., Shahab L., Michie S. Behavior change techniques used by the English stop smoking services and their associations with short‐term quit outcomes. Nicotine Tob Res 2010; 12: 742–747. 20478957 10.1093/ntr/ntq074

[21] West R., Evans A., Michie S. Behavior change techniques used in group‐based behavioral support by the English stop‐smoking services and preliminary assessment of association with short‐term quit outcomes. Nicotine Tob Res 2011; 13: 1316–1320. 21742650 10.1093/ntr/ntr120

[22] DiClemente C. C., Prochaska J. O., Fairhurst S. K., Velicer W. F., Velasquez M. M., Rossi J. S. The process of smoking cessation: an analysis of precontemplation, contemplation, and preparation stages of change. J Consult Clin Psychol 1991; 59: 295–304. 2030191 10.1037//0022-006x.59.2.295

[23] World Health Organization . Toolkit for delivering the 5A's and 5R's brief tobacco interventions in primary care [internet] 2014 [cited 2020 Sep 29]. Available at: http://www.who.int/tobacco/publications/smoking_cessation/9789241506953/en/ (accessed 29 September 2020).

[24] West R., Hajek P., Stead L., Stapleton J. Outcome criteria in smoking cessation trials: proposal for a common standard. Addiction 2005; 100: 299–303. 15733243 10.1111/j.1360-0443.2004.00995.x

[25] Crane D., Ubhi H. K., Brown J., West R. Relative effectiveness of a full versus reduced version of the ‘smoke free’ mobile application for smoking cessation: a randomised controlled trial. F1000Research 2018; 7: 1524. 30728950 10.12688/f1000research.16148.1 PMC6347038

