# Supplementary material for: Effectiveness of training stop‐smoking advisers to deliver cessation support to the UK national proposed standard versus usual care in Malaysia: a two‐arm cluster‐randomized controlled trial
Source: Addiction. 2021 Jan 20;116(8):2150–61. doi: 10.1111/add.15346 (PMC8359305; doi:10.1111/add.15346)
Supplement: Supplementary file 2 — Table S1 Rate of recruitment relative to reported throughput of smokers in the 6 months prior to the study Table S2 Unadjusted and adjusted generalised estimating equations of treatment effect on CO verified continuous abstinence at 4 weeks, 3 months and 6 months Table S3 Predictors of response to 6‐month follow‐up: multivariable logistic regression. [file ADD-116-2150-s001.docx]

**Table 1:  Rate of recruitment relative to reported throughput of smokers in the 6 months prior to the study**

| **Group** | **Smokers attending service in the 6 months prior to the study, *n*** | **Smokers recruited into the study, *n*** | **Ratio of smokers recruited to past-6-month throughput** |
| --- | --- | --- | --- |
| Control | 50 | 4 | 0.08 |
| Control | 60 | 9 | 0.15 |
| Control | 68 | 11 | 0.16 |
| Control | 43 | 13 | 0.30 |
| Control | 32 | 19 | 0.59 |
| Control | 40 | 24 | 0.60 |
| Control | 40 | 32 | 0.80 |
| Control | 20 | 17 | 0.85 |
| Control | 30 | 43 | 1.43 |
| Intervention | 60 | 5 | 0.08 |
| Intervention | 42 | 15 | 0.36 |
| Intervention | 20 | 13 | 0.65 |
| Intervention | 49 | 33 | 0.67 |
| Intervention | 30 | 20 | 0.67 |
| Intervention | 120 | 81 | 0.68 |
| Intervention | 55 | 38 | 0.69 |
| Intervention | 30 | 25 | 0.83 |
| Intervention | 20 | 49 | 2.45 |
| Intervention | 20 | 51 | 2.55 |

**Table 2:  Unadjusted and adjusted generalised estimating equations of treatment effect on CO verified continuous abstinence at 4 weeks, 3 months and 6 months**

|  | **Unadjusted model** | | | **Minimally adjusted model*** | | | **Fully adjusted model**** | | |
| --- | --- | --- | --- | --- | --- | --- | --- | --- | --- |
| **Analysis** | **OR** | **95% CI** | ***p*** | **OR** | **95% CI** | ***p*** | **OR** | **95% CI** | ***p*** |
| **Intention-to-treat** |  |  |  |  |  |  |  |  |  |
| 4 weeks | 2.10 | 1.07-4.13 | 0.032 | 2.20 | 1.01-4.81 | 0.048 | 3.62 | 1.53-8.59 | 0.003 |
| 3 months | 4.17 | 1.37-12.73 | 0.012 | 5.74 | 1.55-21.19 | 0.009 | 8.44 | 1.50-47.63 | 0.016 |
| 6 months | 3.84 | 1.29-11.44 | 0.016 | 4.08 | 1.29-12.88 | 0.017 | 7.89 | 1.28-48.49 | 0.026 |
| **Follow-up only** |  |  |  |  |  |  |  |  |  |
| 4 weeks | 0.69 | 0.29-1.61 | 0.386 | 0.58 | 0.22-1.49 | 0.254 | 0.69 | 0.24-2.01 | 0.499 |
| 3 months | 1.46 | 0.39-5.38 | 0.574 | 1.24 | 0.26-5.80 | 0.789 | 1.43 | 0.19-10.57 | 0.427 |
| 6 months | 1.42 | 0.38-5.27 | 0.601 | 1.33 | 0.33-5.33 | 0.683 | 0.77 | 0.07-8.07 | 0.825 |

CO = carbon monoxide; OR = odds ratio; CI = confidence interval; ICC = intraclass correlation.
* Adjusted for age, sex, education, perceived health problems, and quit method.
** Adjusted for age, sex, ethnicity, education, marital status, occupation, shift work, work stress, perceived health problems, age of smoking initiation, time to first cigarette after waking, cigarettes smoked per day, urges to smoke, smoking at home, exposure to second-hand smoking at home, quit method, baseline CO, motives for smoking, reasons for quitting, confidence to stop smoking completely, and motivation to stop smoking completely.
All models account for clustering.

**Table 3. Predictors of response to 6-month follow-up: multivariable logistic regression**

| **Characteristics** | **OR [95% CI]** |
| --- | --- |
| **Age (years)** |  |
| <25 | 1 |
| 25 - 34 | 1.30 [0.24-6.98] |
| 35 - 44 | 1.01 [0.17-6.07] |
| 45 - 54 | 0.69 [0.11-4.26] |
| >54 | 2.54 [0.39-16.57] |
| **Gender** |  |
| Male | 1 |
| Female | 0.80 [0.12-5.66] |
| **Ethnicity** |  |
| Malay | 1 |
| Chinese | 0.91 [0.39-2.13] |
| Indian | 0.75 [0.28-1.98] |
| Other | 2.13 [0.55-8.29] |
| **Highest level of education** |  |
| None/primary school | 1 |
| Lower/higher secondary school | 0.72 [0.28-1.84] |
| Pre-University/Matriculation/A-Level /Cert/Diploma/Degree | 0.53 [0.17-1.64] |
| **Marital status** |  |
| Unmarried (single/divorced) | 1 |
| Married | 1.42 [0.56-3.59] |
| **Occupation** |  |
| Government | 1 |
| Private | 0.77 [0.31-1.93] |
| Self-employed | 0.47 [0.16-1.35] |
| Other (pensioner/student/housewife/ not working) | 0.35 [0.10-1.22] |
| **Shift work** |  |
| No | 1 |
| Yes | 0.78 [0.35-1.73] |
| **Work stress level** |  |
| No stress | 1 |
| Less stress | 0.90 [0.41-1.97] |
| Stressed | 0.81 [0.33-2.00] |
| Very stressed | 1.66 [0.33-8.23] |
| Not sure | 0.64 [0.17-2.45] |
| **Perceived health problems** |  |
| No | 1 |
| Yes | 0.54 [0.22-1.33] |

| **Age of smoking initiation (years)** | 0.98 [0.91-1.05] |
| --- | --- |
| **Time to first cigarette after waking (minutes)** | |
| > 60 | 1 |
| 31-60 | 1.33 [0.52-3.36] |
| 6-30 | 0.74 [0.30-1.81] |
| ≤ 5 | 0.63 [0.20-1.95] |
| **No. of cigarettes smoked per day** | 0.98 [0.94-1.01] |
| **Urge to smoke in the past 7 days** | |
| Not at all | 1 |
| A little of the time | 2.93 [0.57-14.93] |
| Some of the time | 1.52 [0.32-7.32] |
| Almost all the time | 0.86 [0.17-4.34] |
| All the time | 1.15 [0.20-6.62] |
| **Smokes at home** |  |
| No | 1 |
| Yes | 0.58 [0.31-1.10] |
| **Exposed to second-hand smoke at home** | |
| No | 1 |
| Yes | **0.41 [0.18-0.91]** |
| **Method of quitting** |  |
| Abrupt cessation | 1 |
| Gradual cessation^#^ | 0.60 [0.28-1.30] |
| **CO level** | 1.04 [0.98-1.10] |
| **Motives for smoking** |  |
| Staying calm in stressful situations | 0.97 [0.44-2.12] |
| Keeping you from getting too fat | 0.60 [0.28-1.29] |
| Helping you to concentrate and stay alert | 0.35 [0.09-1.33] |
| Stopping you from being bored | 1.36 [0.65-2.87] |
| Enjoying being with friends | **0.47 [0.24-0.93]** |
| Feeling better when bad things happen | 0.92 [0.45-1.88] |
| Feeling uncomfortable if not smoking | 1.31 [0.68-2.52] |
| Smoking is an enjoyment | 1.14 [0.52-2.47] |
| Other | 0.45 [0.06-3.64] |
| **Reasons to quit smoking** | |
| Pressure from friends and family | 1.47 [0.60-3.58] |
| Concern about personal health/illness | 1.17 [0.54-2.54] |
| Concern about health of other family members | 1.49 [0.72-3.07] |
| Doctor’s/health professional advice and orders | 1.23 [0.58-2.61] |
| Cost of cigarettes increasing (financial) | 0.92 [0.40-2.12] |
| Restriction on smoking in public places | **5.60 [1.30-24.12]** |
| Social stigma (seen in negative light) | 0.75 [0.22-2.52] |
| Religion/beliefs | 1.69 [0.62-4.58] |
| Other | 0.60 [0.00-191.30] |
| **Confidence to stop smoking completely** | |
| Very confident | 1 |
| Quite confident | 2.15 [0.83-5.59] |
| Not very confident | 1.24 [0.30-5.17] |
| Not at all confident | - |
| Not sure | 4.83 [0.35-66.17] |
| **Motivation to stop smoking completely** | |
| Very strong | 1 |
| Quite strong | **0.25 [0.09-0.67]** |
| Not strong | 0.28 [0.07-1.08] |
| Not sure | **0.02 [0.00-0.60]** |
| **Hospital (cluster variable)** | **7.71 [2.72-21.84]** |

CI = confidence interval; OR = odds ratio. Results are from a multivariable model including all variables in the table; model accounts for clustering. Bold font indicates significant results at p<0.05. ^#^Aiming to reduce the number of cigarettes smoked per day over a period of time to 0.
